# Cytotoxicity of Brazilian plant extracts against oral microorganisms of interest to dentistry

**DOI:** 10.1186/1472-6882-13-208

**Published:** 2013-08-15

**Authors:** Jonatas Rafael de Oliveira, Vinicius Carlos de Castro, Polyana das Graças Figueiredo Vilela, Samira Esteves Afonso Camargo, Cláudio Antonio Talge Carvalho, Antonio Olavo Cardoso Jorge, Luciane Dias de Oliveira

**Affiliations:** 1 Department of Biosciences and Oral Diagnosis, Laboratory of Microbiology and Immunology, Institute of Science and Technology, Univ Estadual Paulista/UNESP, Av. Engenheiro Francisco José Longo, 777 - Jardim São Dimas, São José dos Campos, SP, Brazil; 2 Department of Biosciences and Oral Diagnosis, Laboratory of Biochemistry and Pharmacology, Institute of Science and Technology, Univ Estadual Paulista/UNESP, São José dos Campos, SP, Brazil; 3 Department of Restorative Dentistry, Institute of Science and Technology, Univ Estadual Paulista/UNESP, São José dos Campos, SP, Brazil

**Keywords:** *Equisetum arvense* L, *Glycyrrhiza glabra* L, *Punica granatum* L, *Stryphnodendron barbatimam* Mart, *Staphylococcus aureus*, *Staphylococcus epidermidis*, *Streptococcus mutans*, *Candida albicans*, *Candida tropicalis*, *Candida glabrata*

## Abstract

**Background:**

With the emergence of strains resistant to conventional antibiotics, it is important to carry studies using alternative methods to control these microorganisms causing important infections, such as the use of products of plant origin that has demonstrated effective antimicrobial activity besides biocompatibility. Therefore, this study aimed to evaluate the antimicrobial activity of plant extracts of *Equisetum arvense* L., *Glycyrrhiza glabra* L., *Punica granatum* L. and *Stryphnodendron barbatimam* Mart. against *Staphylococcus aureus*, *Staphylococcus epidermidis*, *Streptococcus mutans*, *Candida albicans*, *Candida tropicalis*, and *Candida glabrata*, and to analyze the cytotoxicity of these extracts in cultured murine macrophages (RAW 264.7).

**Methods:**

Antimicrobial activity of plant extracts was evaluated by microdilution method based on Clinical and Laboratory Standards Institute (CLSI), M7-A6 and M27-A2 standards. The cytotoxicity of concentrations that eliminated the microorganisms was evaluated by MTT colorimetric method and by quantification of proinflammatory cytokines (IL-1β and TNF-α) using ELISA.

**Results:**

In determining the minimum microbicidal concentration, *E. arvense* L., *P. granatum* L., and *S. barbatimam* Mart. extracts at a concentration of 50 mg/mL and *G. glabra* L. extract at a concentration of 100 mg/mL, were effective against all microorganisms tested. Regarding cell viability, values were 48% for *E. arvense* L., 76% for *P. granatum* L, 86% for *S. barbatimam* Mart. and 79% for *G. glabra* L. at the same concentrations. About cytokine production after stimulation with the most effective concentrations of the extracts, there was a significant increase of IL-1β in macrophage cultures treated with *S. barbatimam* Mart. (3.98 pg/mL) and *P. granatum* L. (7.72 pg/mL) compared to control (2.20 pg/mL) and a significant decrease of TNF-α was observed in cultures treated with *G. glabra* L. (4.92 pg/mL), *S. barbatimam* Mart. (0.85 pg/mL), *E. arvense* L. (0.83 pg/mL), and *P. granatum* L. (0.00 pg/mL) when compared to control (41.96 pg/mL).

**Conclusions:**

All plant extracts were effective against the microorganisms tested. The *G. glabra* L. extract exhibited least cytotoxicity and the *E. arvense* L. extract was the most cytotoxic.

## Background

Herbal drugs have been used since ancient times as remedies for the treatment of a variety of diseases. Despite advances in modern medicine, plants still make an important contribution to health care [1]. Herbal extracts or essential oils prepared from medicinal plants contain different compounds with numerous biological activities confirmed by *in vitro* and *in vivo* studies, such as antibacterial, antifungal, antiviral, antiprotozoal, anthelminthic, antiseptic, anti-inflammatory, antitumor, antioxidative, antiallergic, anticonvulsant, antidepressant, contraceptive, antimutagenic, analgesic, and diuretic properties [2-4]. Some compounds are used for the treatment of diabetes, nervous system disorders such as Alzheimer’s disease, dental diseases, male infertility, and erectile dysfunction [5], among other therapeutic properties.

Studies have demonstrated the antimicrobial activity of *Equisetum arvense* L. (Equisetaceae), *Glycyrrhiza glabra* L. (Fabaceae), *Punica granatum* L. (Punicaceae), and *Stryphnodendron barbatimam* Mart. (Leguminosae) [6-11]. However, knowledge of the toxicity of these extracts is scarce. Scientific knowledge of the antimicrobial properties of plant species may contribute to the development of drugs against microorganisms of medical interest, particularly in dentistry. In this respect, the environmental conditions of the oral cavity favor infection and inflammation caused by opportunistic microorganisms such as *Staphylococcus* spp., *Streptococcus* spp., and *Candida* spp.

*Staphylococcus aureus* can cause endodontic infections, parotid gland infections, mandibular osteomyelitis, mucositis, apical abscesses, and postoperative complications after dental implant placement [12-14]. Moreover, methicillin-resistant *S. aureus* (MRSA) have emerged as causative agents of serious infections in hospitals [15].

*Staphylococcus epidermidis* is an important agent of hospital-acquired infections and possesses pathogenic properties such as adhesion to implanted materials and biofilm formation [16]. Microorganisms organized in biofilms are more resistant to antimicrobial agents and components of host immunity [17,18].

*Streptococcus mutans* is an important etiological agent of dental caries in humans that attaches to the tooth surface and forms a biofilm [13,14]. For this purpose, the microorganism produces glucosyltransferases and synthesizes an adherent and water-insoluble glucan from sucrose that permits it to adhere firmly to the tooth surface [19]. The colonization of the oral cavity with *S. mutans* depends on factors such as salivary flow, saliva buffering capacity, and presence of salivary immunoglobulins [20].

Biofilm formation is an important virulence factor for *Candida* species because it increases resistance to antifungal therapy [21,22]. *C. albicans* is the most pathogenic species in humans and is commonly identified in dental prostheses and oral candidiasis. These yeasts colonize the oral mucosa, tongue and palate. Oral, vaginal or systemic candidiasis is a common infection in immunocompromised patients that can be fatal [13,14,21,23,24].

Alternative microbiological control measures that are suitable for use in humans are of the utmost importance in view of the emergence of antimicrobial-resistant strains. Therefore, the present study evaluated the effectiveness of herbal extracts of *E. arvense* L., *G. glabra* L., *P. granatum* L. and *S. barbatimam* Mart. against clinical strains of *Staphylococcus* spp.*, S. mutans* and *Candida* spp. In addition, the cytotoxic effects of the extract were investigated based on cell viability and cytokine production (interleukin 1β [IL-1β] and tumor necrosis factor α [TNF-α]) in cultured mouse macrophages (RAW 264.7).

## Methods

The study was approved by the Ethics Committee of the São José dos Campos Dental School, UNESP (protocol 008/2012-PA/CEP).

### Antimicrobial activity

Dry powders of *E. arvense* L., *G. glabra* L., *P. granatum* L. and *S. barbatimam* Mart. were purchased from the Oficina de Ervas (Ribeirão Preto, São Paulo, Brazil) and the extracts were prepared in propylene glycol (200 mg/mL).

Nine clinical strains and reference strains (ATCC) of *S. aureus* (ATCC 6538), *S. epidermidis* (ATCC 12228), *S. mutans* (ATCC 35688), *C. albicans* (ATCC 18804), *C. tropicalis* (ATCC 13803) and *C. glabrata* (ATCC 90030) obtained from the Laboratory of Microbiology and Immunology, Institute of Science and Technology, were used. The clinical strains were identified after isolation from the oral cavity of patients with pulmonary tuberculosis. A total of 60 strains were tested.

Bacteria and yeast were cultured in brain-heart infusion (BHI – Himedia, Mumbai, Maharashtra, India) and Sabouraud-dextrose broth (Himedia), respectively, for 24 h at 37°C. *S. mutans* was incubated under microaerophilic conditions (5% CO_2_). The microbial suspensions were prepared in sterile saline (0.9% NaCl) at a standard concentration of 5 × 10^5^ cells/mL for bacteria and 1 × 10^3^ to 5 × 10^3^ cells/mL for yeast.

The microdilution method was performed according to NCCLS guidelines [25,26], changes name to Clinical and Laboratory Standards Institute (CLSI). For this purpose, 10 dilutions of the plant extracts (100 to 0.19 mg/mL) were prepared in Mueller-Hinton medium (Himedia) for bacteria and in RPMI 1640 medium (Himedia) plus MOPS buffer (Sigma Aldrich, St. Louis, Missouri, USA), pH 7.0 ± 0.1, for yeast. Next, 5 μL of the bacterial suspension or 100 μL of the yeast suspension was inoculated into each well of 96-well plates (TPP, Trasadingen, Schaffhausen, Switzerland) and the plates were incubated for 24 h at 37°C (under microaerophilic conditions for *S. mutans*).

After determination of the Minimum Inhibitory Concentration (MIC), 100 μL of this concentration and one above and one below this concentration were seeded onto BHI or Sabouraud-dextrose agar plates to determine the Minimum Microbicidal Concentration (MMC) of the extracts. After 48 h of incubation, the MMC was determined on plates that showed no growth of colonies. The results are reported as the percentage of strains inhibited at each concentration of the plant extract.

### Cell culture

Mouse macrophages (RAW 264.7) obtained from the Rio de Janeiro Cell Bank - Associação Técnico Científica Paul Ehrlich (APABCAM - Rio de Janeiro, RJ, Brazil) were cultured routinely in Dulbecco’s Modified Eagle Medium (DMEM - LGC Biotechnology, Cotia, SP, Brazil) supplemented with 10% fetal bovine serum (FBS – Gibco, USA) at 37°C in a 5% CO_2_ atmosphere. Viable cells were counted by the Trypan blue (0.5%, Sigma-Aldrich, St. Louis, MO, USA) exclusion method.

### Cytotoxicity testing (MTT assay)

For the experimental and positive control groups, 8 × 10^3^ viable cells/well were seeded in 96-well plates (Nunc, Kamstrupvej, Roskilde, Denmark) and the plates were incubated for 24 h at 37°C. Next, the cell cultures were exposed to 200 μL of the serial dilutions of the plant extracts according to the concentrations obtained in the microbiological test and incubated for 24 h. At the end of exposure, the cell culture medium was discarded and cell survival was determined by the MTT assay [MTT - (3-(4,5-dimethylthiazol-2-yl)-2,5-diphenyltetrazolium bromide; Sigma-Aldrich].

The plates were washed with phosphate-buffered saline (PBS - Cultilab, Campinas, São Paulo, Brazil) and 100 μL MTT solution (0.5 mg/mL in PBS) was added to each well. After incubation for 1 h, MTT solution was removed and 100 μL dimethyl sulfoxide (DMSO - Sigma-Aldrich) was added to each well. The plates were incubated for 10 min and then shaken on a shaker for an additional 10 min. Optical densities were measured in a multi-well spectrophotometer (Bio-Tek, Winooski, Vermont, USA) at 570 nm. The optical density values obtained for cultures exposed to the extracts were normalized to untreated control cultures (corresponding to 100%).

The results of the cytotoxicity tests were analyzed by ANOVA and the Tukey test (P ≤ 0.05) using the BioEstat 5.0 software.

### Quantification of IL-1β and TNF-α

For analysis of cytokine production, 1 × 10^6^ RAW264.7 cells were seeded in 24-well plates and incubated for 24 h at 37°C. Next, the cell cultures were exposed to concentrations of the plant extracts that were the most effective in the microbiological test. After incubation for 24 h, the supernatant was collected from each well and frozen at −18°C.

The levels of IL-1β and TNF-α in the supernatants were determined by enzyme-linked immunosorbent assay (ELISA) using the DuoSet ELISA Development System for IL-1β (DY401) and TNF-α (DY410) ((R&D Systems, Minneapolis, Minnesota, USA) according to manufacturer instructions. Optical densities were read on a microplate spectrophotometer at 450 nm and converted into IL-1β and TNF-α concentrations (pg/mL) using the GraphPad Prism 5.0 program.

The results were analyzed by ANOVA and the Tukey test (P ≤ 0.05) using the BioEstat 5.0 software.

## Results

The antimicrobial activity of the plant extracts is shown in Table 1. The *E. arvense* L. extract, at a concentration of 50 mg/mL, eliminated 100% of *S. aureus*, *S. epidermidis*, *C. albicans*, *C. tropicalis*, and *C. glabrata* strains. At a concentration of 25 mg/mL, this extract eliminated 100% of *S. mutans.* The MMC of the *G. glabra* L. extract was 100 mg/mL for *S. aureus*, *S. epidermidis* and *S. mutans*, and 50 mg/mL for yeast (*C. albicans*, *C. tropicalis*, and *C. glabrata*). The *P. granatum* L. extract presented an MMC of 25 mg/mL for *S. aureus* and *S. epidermidis*, 12.5 mg/mL for *S. mutans*, and 50 mg/mL for yeast. The widest variation in MMC was observed for the *S. barbatimam* Mart. extract, with an MMC of 3.13 mg/mL for *S. mutans*, 12.5 mg/mL for *S. epidermidis*, 25 mg/mL for *S. aureus*, and 50 mg/mL for yeasts.

**Table 1:** Values of the MMC (mg/mL) of plant extracts for all microorganisms evaluated

Microorganism*	**Plant extract**			
	***E. arvense *L.**	***G. glabra *L.**	***P. granatum *L.**	***S. barbatimam *Mart.**
*S. aureus*	50	100	25	25
*S. epidermidis*	50	100	25	12.5
*S. mutans*	25	100	12.5	3.13
*C. albicans*	50	50	50	50
*C. tropicalis*	50	50	50	50
*C. glabrata*	50	50	50	50

*Ten strains of each microbial species were evaluated.

Cytotoxicity of the plant extracts was evaluated in cultured macrophages by the MTT assay. The highest cell survival rates were observed for cultures treated with *G. glabra* L., *P. granatum* L. and *S. barbatimam* Mart. The group treated with the *E. arvense* L. extract exhibited the lowest survival rate (<50%) (Table 2).

**Table 2:** Percentage of cell viability (± SD) after treatment with the MMC of plant extracts

**Concentration (mg/mL)**	**Plant extract**			
	***E. arvense *L.**	***G. glabra *L.**	***P. granatum *L.**	***S. barbatimam *Mart.**
100	-	79 ± 6.18^B^	-	-
50	48 ± 5.29^B^	52 ± 2.96^C^	76 ± 3.10^B^	86 ± 4.05^B^
25	45 ± 1.08^B^	-	57 ± 5.24^C^	70 ± 4.96 ^C^
12,5	-	-	57 ± 3.70^C^	65 ± 4.91^C^
3,13	-	-	-	72 ± 6.27^C^
*Control*	100^A^	100^A^	100^A^	100^A^

Different superscript letters indicate statistically significant differences (P < 0.05).

Analysis of cytokine production showed a significant increase of IL-1β production in cultures treated with the *P. granatum* L. and *S. barbatimam* Mart. extracts when compared to the control group. No difference in IL-1β production was observed between cultures treated with the *E. arvense* L. and *G. glabra* L. extracts and the control group (Table 3).

**Table 3:** **Levels of IL-1β and TNF-α (mean ± standard deviation, pg/mL) in macrophage culture supernatants after exposure to plant extracts (100 mg/mL for *G. glabra *L. and 50 mg/ml for *E. arvense *L., *P. granatum *L. and *S. barbatimam *Mart.)**

**Plant extract**	**Cytokine production (pg/mL)**	
	**IL-1β**	**TNF-α**
*E. arvense* L.	1.32 ± 1.37^A^	0.83 ± 2.86^B^
*G. glabra* L.	1.99 ± 1.46^A^	4.92 ±1.46^B^
*P. granatum* L.	7.72 ±1.13^C^	0.00^B^
*S. barbatimam* Mart.	3.98 ± 1.40^B^	0.85 ± 2.93^B^
*Control*	2.20 ± 2.11^A^	41.96 ± 10.53^A^

Different superscript letters indicate statistically significant differences (P < 0.05).

All extracts significantly reduced the production of TNF-α when compared to the control group, with complete inhibition of TNF-α production in the *P. granatum* L.-treated group (Table 3).

## Discussion

The present study evaluated the antimicrobial activity of plant extracts not only against reference strains (ATCC), but also against clinical isolates since differences in antimicrobial susceptibility exist between the same species isolated from different patients as demonstrated by the present results. The four extracts exerted microbicidal activity against six microbial species. However, the MMC varied according to the extract and its concentration used and according to bacterial or fungal species studied.

The *S. mutans* isolates showed high sensitivity to the *E. arvense* L. extract, with a concentration of 25 mg/mL inhibiting the growth of all strains. At a concentration of 50 mg/mL, *E. arvense* L. was effective against all *Staphylococcus* spp. and *Candida* spp. isolates. The antimicrobial activity of *E. arvense* L. can be attributed to the presence of various compounds such as the phenolic monoterpene thymol, capable inhibit the growth of microorganisms [8,27]. It was reported that concentrations of *E. arvense* L. hydromethanolic extracts presented a dose-dependent activity in *S. aureus*, at 10–1000 mg/mL, showing a total elimination at 1000 mg/mL [28]. According to Radulovic et al. [27] the essential oil of *E. arvense* L. also showed activity against *S. aureus* and *C. albicans* strains, in agreement with our study, yet it was effective against Gram-negative bacteria *Klebsiella pneumoniae*, *Pseudomonas aeruginosa*, *Salmonella enteritidis*, *Escherichia coli*, and against *Aspergillus niger*. In vitro tests also demonstrated the inhibitory effect of *E. arvense* L. hydroethanolic extract against *Neisseria gonorrhoeae*[29].

The *G. glabra* L. extract inhibited the growth of all bacterial strains studied at a concentration of 100 mg/mL. In contrast, fungal growth was inhibited at a concentration of less than 50 mg/mL, demonstrating that *Candida* spp. were more sensitive to the extract than the bacterial strains. Antifungal activity of this plant was attributed to the presence of glabridin, hispaglabridin B [30] and 18β-glycyrrhetinic acid [9]. Glabridin exhibiting inhibitory activity against *C. albicans* strain and inhibited the growth of this yeast mutant strain resistant to amphotericin B, nystatin and clotrimazole [30]. This compound also showed inhibitory effect on the growth of *S. aureus* strains sensitive and resistant to methicillin, showing MIC at 12.5 mg/mL [31]. The 18β-glycyrrhetinic acid caused inhibitory effect for *C. albicans* at concentrations of 50 and 100 mg/mL [9]. Clinical and reference strains of this yeast was also proven their susceptibility to the extract of *G. glabra* L. prepared in different solvents as ethanol, ethyl acetate, and hexane [32]. According to our study, Hwang et al. [33] also found the antibacterial extract *G. glabra* L regard to *S. mutans*. Statti et al. [34] collected *G. glabra* L. from different regions of Calabria, Italy, and verified an inhibitory effect on bacteria and fungi, and this biological activity varied due to differences in the chemical composition of this plant obtained from different sites.

The *P. granatum* L. extract completely inhibited the growth of *S. mutans*, *Staphylococcus* spp. and *Candida* spp. at concentrations of 12.5, 25 and 50 mg/mL, respectively. Thus, *S. mutans* showed high sensitivity to the extract, whereas *Candida* spp. exhibited low sensitivity to *P. granatum* L., requiring higher concentrations for growth inhibition. Rosas-Piñón et al. [35] studying the medicinal plants from a Mexico region used by local people to treat dental diseases such as toothache, dental caries, periodontal disease and gingivitis, found the antimicrobial activity of 47 plant species on *Porphyromonas gingivalis* and *S. mutans*. According to our study, the authors verified that *S. mutans* was more sensitive to *P. granatum* L extract, compared to *P. gingivalis*, showing lower inhibitory concentrations of growth. Thus, it is believed that this higher sensitivity of streptococcal is related to its cell composition. Products based on *P. granatum* L., as commercial sauces and juices also showed antimicrobial activity against natural microbiota of vegetables such as lettuce, chives and parsley, as well as on *S. aureus* and *E. coli* that was inoculated on these plants [36]. Our results are in agreement with Abdollahzadeh et al. [37], which verified that *P. granatum* L. extract was effective on oral opportunistic pathogens such as *S. aureus*, *S. epidermidis*, *S. mutans* and *C. albicans*. Also, Fawole et al. [38] showed that *P. granatum* L. extract was effective on *S. aureus*, and this extract also presented antimicrobial activity against *K. pneumoniae* and *E. coli*. Some studies have also been proven the effectiveness of *P. granatum* L. against MRSA, *Listeria monocytogenes*, *Bacillus subtilis*, *S. enteritidis* and *Yersinia enterocolitica*[39]. The bactericidal activity of this plant is due to the presence of tannins and punicalagins [40,41]. A paste based on the extract of *P. granatum* L. [42] was produced and applied on wounds created in guinea pigs and their potential healing was confirmed with total regression of the wound in 20 days, and also effectively control the microbiota of the wound. In vitro tests have demonstrated the antimicrobial activity of this extract on *S. aureus*, *P. aeruginosa*, *E. coli*, *K. pneumoniae*, *Salmonella anatum*, *S. typhimurium*, *S. pneumoniae*, *C. albicans*, *C. glabrata*, and *A. rubrum Trichopyton niger*. Vasconcelos et al. [7] also used a gel based on *P. granatum* L. extract to control *S. mutans*, *S. sanguis*, *S. mitis* and *C. albicans*, alone or associated with each other, and it has shown inhibition of adhesion of these microorganisms on the glass surface. By inhibiting the adherence of *S. mutans* other species also have their adhesion impaired, since this streptococci fail to provide the support to anchor these pathogens; e.g., *C. albicans* on the tooth surface [43].

The *S. barbatimam* Mart. extract exerted microbicidal activity at different concentrations, which were 3.13 mg/mL for *S. mutans*, 12.5 mg/mL for *S. aureus*, and 25 mg/mL for S*. epidermidis*. However, only the concentration of 50 mg/mL was effective against the *Candida* species tested. The results of our study demonstrate that *S. mutans* was the most sensitive specie to *S. barbatimam* Mart. extract, and *C. albicans* was less sensitive, agreeing with the study by Pereira et al. [44], which the extract of *Stryphnodendron* spp. also had lower and higher MMC against *S. mutans* and *C. albicans*, respectively. The extract of *Stryphnodendro*n spp. produced in aqueous and ethyl-acetate fractions showed significant antifungal activity against clinical isolates of *C. albicans*, *C. parapsilosis* and *C. tropicalis*, and revealed that the presence of tannins affected the integrity of the cell wall of *Candida*, a factor that contributes to decreased adhesion to host cells and germ-tube formation, besides affecting the budding process and stimulate phagocytosis [6].

The present study also analyzed the cytotoxicity of the *E. arvense* L., *G. glabra* L., *P. granatum* L. and *S. barbatimam* Mart extracts in macrophage cultures (RAW 264.7) using the most effective concentrations obtained in the microbiological test. Except for *P. granatum* L., there are no studies in the literature investigating the cytotoxicity of these plants by the methods used here. After exposure for 24 h to *G. glabra* L., at concentrations of 100 and 50 mg/mL, reduced cell survival by 79% (± 6.18) and 52% (± 2.96), respectively. The viability of cells treated with *P. granatum* L. at a concentration of 50 mg/mL was 76% (± 3.10), whereas the percentage of surviving cells was 57% (± 5.24) and 57% (± 3.70) at concentrations of 25 and 12.5 mg/mL, respectively. The percentage of surviving cells was 86% (± 4.05), 70% (± 4.96), 65% (± 4.91), and 72% (± 6.27) for macrophages treated with *S. barbatimam* Mart. at concentrations of 50, 25, 12.5 and 3.13 mg/mL, respectively. Cell viability was less than 50% only in the groups treated with *E. arvense* L., i.e., 48% (± 5.29) at 50 mg/mL and 45% (± 1.08) at 25 mg/mL. It has been demonstrated reduction in cell viability after 24 h exposure to antimicrobials used in endodontic therapy such as ciprofloxacin hydrochloride, clindamycin hydrochloride, and metronidazole, with these drugs, showing dose-dependent cytotoxicity [45]. In the present study, cell viability was significantly higher in the groups treated with higher concentrations of *G. glabra* L., *P. granatum* L. and *S. barbatimam* Mart. when compared to the groups treated with lower concentrations.

The cytotoxicity of the plant extracts was also investigated by quantifying the production of IL-1β and TNF-α in cell cultures (RAW 264.7) after 24-h exposure to concentrations of the extracts that were most effective against the microorganisms tested. No significant difference in the production of IL-1β was observed between cultures treated with *E. arvense* L. (1.32 ± 1.37 pg/mL) and *G. glabra* L. (1.99 ± 1.46 pg/mL) and the control group (2.20 ± 2.11 pg/mL). In contrast, production of this cytokine was significantly higher in cultures treated with *S. barbatimam* Mart. (3.98 ± 1.40 pg/mL) and *P. granatum* L. (7.72 ± 1.13 pg/mL). The production of TNF-α was significantly lower in cultures treated with *E. arvense* L. (0.83 ± 2.86 pg/mL), *G. glabra* L. (4.92 ± 1.46 pg/mL) and *S. barbatimam* Mart. (0.85 ± 2.93 pg/mL) when compared to the group not stimulated with the extracts (41.96 ± 10.53 pg/mL). The *P. granatum* L. extract completely inhibited the synthesis of TNF-α. Thus, it was observed that the basal levels of TNF-α were higher than those of IL-1β. This behavior also was shown in other studies [46,47].

The study of the cytotoxicity and antimicrobial activity of natural products such as plant extracts is important for the clinical application of these compounds in different areas of health care. For example, in dentistry, mouthwashes, root canal irrigants, intracanal medications and even toothpastes with antimicrobial activity could be developed that are safe for use in humans. However, animal studies and subsequent clinical trials are needed to confirm the safety of these products.

## Conclusion

In conclusion, the *E. arvense* L., *G. glabra* L. *P. granatum* L. and *S. barbatimam* Mart. extracts exerted microbicidal activity against all *Staphylococcus* spp., *S. mutans* and *Candida* spp. strains tested. The *G. glabra* extract exhibited least cytotoxicity and the *E. arvense* extract was the most cytotoxic.

## Abbreviations

°C: Degrees celsius; ANOVA: Analysis of variance; ATCC: American Type Culture Collection; BHI: Brain-heart infusion; CLSI: Clinical and Laboratory Standards Institute; CO2: Carbon dioxide; DMEM: Dulbecco’s Modified Eagle Medium; DMSO: Dimethyl sulfoxide; ELISA: Enzyme-linked immunosorbent assay; FBS: Fetal bovine serum; h: Hour(s); IL-1β: Interleukin 1-beta; mg/mL: Milligram per milliliter; MIC: Minimum Inhibitory Concentration; MMC: Minimum Microbicidal Concentration; MOPS: 3-(N-morpholino) propanesulfonic acid; MRSA: Methicillin-resistant *S. aureus*; MTT: (3-(4,5-dimethylthiazol-2-yl)-2,5-diphenyltetrazolium bromide; NaCl: Sodium chloride; NCCLS: National Committee for Clinical Laboratory Standards; nm: Nanometers; PBS: Phosphate-buffered saline; pg/mL: Picograms per milliliter; RAW 264.7: Cell line of mouse macrophages; RPMI: Medium developed by Moore et al. in Roswell Park Memorial Institute; TNF-α: Tumor necrosis factor alpha.

## Competing interests

No conflict of interests for this study with any person or institution.

## Authors’ contributions

JRO: conception, design of the experiments, interpretation of data, redaction of the manuscript and attainment of the tables. VCC: design of the experiments. PGFV: conception, design of the experiments. SEAC: redaction and review of the manuscript. CATC: conception and redaction of the manuscript. AOCJ: conception, interpretation of data and redaction of the manuscript. LDO: conception, interpretation of data, critical analysis and redaction of the manuscript. All authors read and approved the final manuscript.

## Pre-publication history

The pre-publication history for this paper can be accessed here:

http://www.biomedcentral.com/1472-6882/13/208/prepub
